# Improvements in Safety Outcomes Following Brief Healthcare-Based Intimate Partner Violence Interventions among Women Who Screen Positive for Intimate Partner Violence-Related Traumatic Brain Injuries

**DOI:** 10.3390/brainsci14101008

**Published:** 2024-10-06

**Authors:** Michelle M. Pebole, Brigitta M. Beck, Colin T. Mahoney, Katherine M. Iverson

**Affiliations:** 1 The Translational Research Center for TBI and Stress Disorders, VA Boston Healthcare System, Boston, MA 02130, USA; 2 Department of Psychiatry, Harvard Medical School, Boston, MA 02215, USA; 3 Department of Psychology, University of Colorado, Colorado Springs, CO 80918, USA; 4 Lyda Hill Institute for Human Resilience, Colorado Springs, CO 80918, USA; 5 Women’s Health Sciences Division, National Center for PTSD, VA Boston Healthcare System, Boston, MA 02130, USA; 6 Department of Psychiatry, Chobanian and Avedisian School of Medicine, Boston University, Boston, MA 02118, USA

**Keywords:** domestic violence, concussion, safety, treatment effectiveness

## Abstract

**Background:** Traumatic brain injuries (TBIs) are a common consequence of experiencing intimate partner violence (IPV). IPV-related TBI contributes to adverse health outcomes among women, but it is unknown whether a history of IPV-related TBI negatively impacts safety outcomes following healthcare-based interventions for IPV. **Methods:** Using data from a larger randomized clinical trial, we explored the impact of IPV-related TBI status on safety-related outcomes in two healthcare-based IPV interventions. At baseline, 35% (*n* = 21) of the sample screened positive for IPV-related TBI history. We used repeated measures ANOVAs to examine differences in safety outcomes at post-treatment and 1-month follow-up based on treatment condition and IPV-related TBI status. **Results:** Significant interaction effects were found for safety outcomes, such that women with IPV-related TBI history experienced larger reductions in the frequency of physical IPV and similar reductions in sexual IPV across both treatment conditions compared to women without IPV-TBI (*F*(2, 102) = 10.88, *p* < 0.001; *F*(2, 98) = 3.93, *p* = 0.036). **Conclusions:** Findings suggest that brief healthcare-based IPV interventions may result in improvements in safety outcomes for women with a history of IPV-TBI. This highlights the continued need for implementation of promising IPV-focused interventions to promote safety and protect women from experiencing further IPV.

## 1. Introduction

Intimate partner violence (IPV) against women, defined as physical, psychological, and/or sexual violence from a current or past partner is a worldwide public health issue [1,2]. Recent data from the National Intimate Partner and Sexual Violence Survey report approximately 41% of women experience IPV during their lifetime [3,4,5]. Further, IPV is often a chronic and recurring experience; between 22 and 46% of women survivors of IPV reported revictimization (i.e., additional victimization) over a 6-month period [6,7]. The prevalence of IPV increased during the COVID-19 pandemic, with some areas in the United States seeing up to a 20% increase [8]. This prevalence highlights the importance of better understanding health and safety outcomes in women impacted by IPV.

IPV is associated with myriad physical and mental health consequences. Nearly half (45%) of women who experience IPV develop post-traumatic stress disorder (PTSD), and frequently experience comorbid mental health issues such as anxiety, depression, low quality of life, substance use disorders, and suicidal ideation [2,5,9,10,11]. Additionally, women survivors of IPV often experience psychosomatic manifestations due to chronic stress, which may present as headaches (including migraines), dizziness [12], digestive complaints (e.g., nausea, abdominal pain) [13], odynophagia (i.e., painful swallowing) [14], and heart palpations [13,14]. Co-occurring physical health concerns such as chronic pain and inflammation, poor sleep, and high risk for cardiometabolic disease also contribute to poor functional status among women who have experienced IPV [15,16,17,18]. Finally, women survivors of IPV are at a heightened risk of engaging in risk-taking behaviors, such as disordered eating patterns, risky sexual behaviors (e.g., multiple partners, improper use of contraception), non-suicidal self-harm and hazardous substance use, which increases the likelihood of serious injury, and even mortality [14,19]. These physical, mental, and behavioral health consequences demonstrate the profound impact IPV can have on women’s lives.

Over 40% of women experience physical IPV and around 32.5% experience severe physical IPV (e.g., being hit with a fist or hard object, strangled, beaten, or assaulted with a weapon) over their lifetime [5]. These assaults to the head, neck, and face often repeat and intensify over time [20,21], substantially increasing risk for traumatic brain injuries (TBIs) among women. TBI, defined as a physiological disruption in brain functioning due to an external force to the head or neck [22], can result in disruptions to cognitive, behavioral, communication, and emotional functioning [23]. These changes in functioning can last f hours or days from the date of the injury and can include ongoing chronic physical and mental health symptoms and conditions [15,24]. Between 33 and 75% of women who experience intimate partner violence (IPV) experience a traumatic brain injury (TBI) from an intimate partner [25,26]. Women who experience physical IPV, including IPV-related TBI, are more likely to be diagnosed with PTSD and experience other psychosocial health risks, such as neurobehavioral symptoms (e.g., dizziness, poor coordination, difficulty with concentration and memory, irritability) and headaches, compared to those who do not experience physical IPV from a partner [27,28]. These conditions can intensify if physical IPV is repeated, and women who live with a violent partner are at an increased risk of repetitive experiences of IPV [18]. Further, women who experience IPV-related TBI also experience contextual factors such as fear [29], coercive control [30], and barriers to accessing care [31], which exacerbate these poor psychosocial outcomes. Despite the increasing awareness of IPV-related TBI and its deleterious impacts on physical and psychosocial functioning, there is limited understanding as to whether a history of IPV-related TBI can impact psychosocial treatment and associated safety-related outcomes for women with histories of IPV.

Trauma-informed interventions are needed in healthcare settings to improve health and well-being among women who experience IPV [32,33]. Most interventions offered in healthcare settings are psychological treatments which target mental health problems. These interventions have moderate evidence indicating their beneficial effects on mental health domains such as depression symptoms [34,35]. Safety outcomes like re-exposure to physical, sexual, or psychological violence and safety planning are also critical for IPV, given that IPV is associated with recurrent physical violence, psychological abuse and coercion [36,37,38,39,40]. Indeed, several interventions aimed at increasing relationship safety have demonstrated improvements in safety and violence reductions [41]; however, these did not examine whether women had a history of IPV-related TBI. More evidence supporting safety among women who have experienced IPV-related TBI would benefit this field. Additionally, previous research demonstrates that a history of TBI may negatively impact treatment effects [42,43,44], although this research does not include IPV samples or focus on safety outcomes. The dearth of evidence examining treatment-related safety outcomes among women who have experienced IPV-related TBI underscores the need for more research in this area. The importance of this work should not be understated, given the high prevalence of IPV-related TBI and associated deleterious physical and mental health sequelae.

Recovering from IPV through Strengths and Empowerment (RISE) is a brief, clinician-administered, survivor-centered counseling intervention rooted in principles of empowerment, trauma-informed care, and motivational interviewing [45,46,47]. This treatment is an individualized, variable-length, and modular intervention that targets key concerns of IPV survivors. RISE has demonstrated improvements in self-efficacy, depression, and valued living, among Veterans experiencing IPV in both clinical and research settings [46,47]. Most recently, RISE was tested against an advocacy-based enhanced-care-as-usual (ECAU) intervention, a single-session advocacy-based approach combining psychoeducation, safety planning, validation, and connection to community resources [45]. Results from this randomized clinical trial (RCT) indicated RISE participants experienced significantly higher increases in empowerment and self-efficacy compared to ECAU, while both groups experienced significant improvements in valued living, depression, and IPV reduction over time [45]. Although these psychosocial benefits are encouraging, the impact of IPV-related TBI on safety outcomes in this RCT has not yet been examined. Understanding the impact of IPV-TBI on IPV-related safety outcomes, such as the frequency of physical and sexual IPV revictimization, would address current gaps in knowledge and would be informative for implementing effective clinical programs inclusive of women with histories of IPV-related TBI.

This study is a secondary analysis of RCT data, aiming to explore the impact of IPV-related TBI status on safety-related outcomes in both RISE and ECAU. Although this study is exploratory, we anticipate that both RISE and ECAU will result in significantly reduced experiences of physical and sexual IPV revictimization for both IPV-related TBI survivors and those without an IPV-TBI history, with RISE showing even stronger effects in comparison to ECAU, given previous treatment differences on self-efficacy and empowerment (which may be particularly relevant for women with a history of IPV-related TBI) [43]. Results may address gaps in knowledge around the impact of IPV-related TBI on treatment and recovery outcomes among women with histories of IPV. This information is essential to understand the impacts that IPV-related TBI may have on health and safety, and can guide potential tailoring of interventions based on history of IPV-TBI.

## 2. Methods and Materials

### 2.1. Study Design

The current study utilized data from a parent RCT, testing RISE against ECAU (in-depth information about the RCT design, RISE, and ECAU can be found elsewhere [45,46,47]).We provide an abbreviated version of the methods here. In brief, Veterans Health Administration (VHA) patients were recruited between October 2018 and September 2020 from the northeastern United States. Inclusion criteria included women who reported (1) experiencing IPV within the past year, including 1 or more incidents of physical, psychological or sexual IPV determined by the Conflict Tactics Scale-Revised (CTS2) [48], (2) receiving care from the VHA within the past year, (3) being at least 18 years or older, and (4) willingness to be audio recorded. Exclusion criteria included current symptoms or diagnosis of bipolar or psychotic disorder and suicidal and/or homicidal ideation with intent. Assessments of safety-related outcomes (i.e., IPV) occurred at baseline, 10 week, and 14 weeks. The study was approved by the VA Boston Healthcare System’s Institutional Review Board, and all participants provided consent (ClinicalTrials.gov identifier: NCT03261700).

### 2.2. Participants and Procedures

Participants were women who reported past-year IPV and received a brief intervention for IPV (RISE or ECAU) within an urban healthcare setting (N = 60). There were no significant differences between participant characteristics at baseline in either the RISE or ECAU conditions [45]. As reported in the parent RCT, on average, participants were in their late thirties (39.1 years; SD = 11.9) with 45% of the sample identifying as a racial minority and more than half of the sample (56.7%) reporting an annual household income over USD 45,000. Approximately half of the participants (45.0%) reported ongoing involvement with a violent partner.

### 2.3. Measures

#### 2.3.1. Participant Characteristics

The participant’s sociodemographic and mental health characteristics were assessed via self-report. Probable mental health conditions were determined using standard clinical cutoffs on the Center for Epidemiological Studies Depression Scale (CES-D; cutoff 16+) [49], the Depression Anxiety Stress Scale–Anxiety Subscale (cutoff 8+) [50,51], and the PTSD Checklist-5 (cutoff 33+) [52,53].

#### 2.3.2. IPV-Related TBI Screening

IPV-related TBI status was evaluated using a modified version of the VA TBI self-report screening tool [54], which has been used to identify probable IPV-related TBI in prior research [55]. Participants screened positive for IPV-TBI if they self-reported experiencing one or more physically violent events and TBI-related symptoms. Types of violent events by an intimate partner included (a) being hit on the head with an object, head, or fist; (b) being pushed or shoved into a wall, car, furniture, or other object; (c) having broken teeth or jaw; (d) having eye or ear injuries; (f) being strangled or choked and (g) other injuries to the head, neck, or face. In alignment with the VA/Department of Defense clinical practice guidelines [56], to screen positive for IPV-related TBI, participants also had to report any of the following symptoms: loss of consciousness (i.e., being unresponsive to the external world), alterations in consciousness (i.e., being dazed or confused or impaired thinking patterns), post-traumatic amnesia (i.e., not remembering the event itself or time before or after the event), and concussion or head injury following an instance of an IPV-related head injury event. Participants screened negative for IPV-TBI if they did not self-report experiencing an IPV-related head injury event or if they self-reported experiencing an IPV-related head injury event but denied experiencing loss of consciousness, alterations in consciousness, post-traumatic amnesia, concussion or head injury following the IPV-head injury event.

### 2.4. Safety Outcomes

Participants’ past year physical and sexual IPV exposure was assessed using the (CTS2) [57], a self-report measure which includes subscales that assess physical (e.g., hitting) and sexual (e.g., rape) IPV experiences. The participants respond to Likert-type questions indicating the number of times they had experienced each type of IPV behavior within the past year. Count scores were calculated for both subscales, with higher scores suggesting greater levels of IPV [58]. The CTS2 has demonstrated construct validity and internal consistency in previous studies [57,59].

### 2.5. Intervention: RISE

RISE is a variable-length psychosocial intervention that included up to 6 sessions over the course of 10 weeks [45]. To promote choice, voice, and empowerment, participants choose from one of six modules to focus on in each session. These modules include Safety Planning, Education on Health Effects of IPV and Warning Signs, Improving Coping and Self-Care, Enhancing Social Support, Making Difficult Decisions, and Connecting with Resources and Moving Forward. Modules include handouts and exercises to promote knowledge and skills building. Clinicians utilize motivational interviewing to help women navigate ambivalence and articulate the importance and confidence of their goals. Modules do not need to be delivered sequentially, nor do they all need to be covered, and women can choose to repeat modules that are most useful to them. At the end of each session, women choose whether to schedule another session. RISE maintains a nonjudgmental, survivor-centered stance. In-depth information about the development, implementation, and effectiveness of RISE are detailed in previous publications [45,46,47].

### 2.6. Intervention: ECAU

The ECAU condition used an advocacy-based approach that incorporates best practices for addressing IPV in VHA. It comprised of a single 60 min session with a provider. During this session, participants received an IPV educational brochure and information on VHA community resources, and were offered safety planning. Guided by the brochure, providers discussed the different forms of IPV and the effects of IPV on physical, mental, and social health. ECAU also included validation through supportive statements (e.g., “I’m sorry this is happening to you”).

### 2.7. Statistical Analyses

We used IBM SPSS Version 29 for this secondary analysis of data from a parent RCT. First, independent *t*-tests and chi-square analyses were conducted to compare baseline differences in demographic variables between women with a history of IPV-related TBI and those without a history of IPV-related TBI. Second, general linear models using repeated-measures ANOVAs were conducted, with treatment condition and IPV-related TBI history as the between-subjects factors and time as the within-subjects factor, with count scores of physical- and sexual-IPV victimization experiences as the primary outcomes. Preliminary analyses suggested that all statistical assumptions were met for these models, except for the assumption of sphericity, which was addressed by using the Greenhouse–Geisser and Huynd–Feldt corrections, based on the estimate of epsilon. We used partial eta squared as a measure of effect size, with 0.01 to less-than or equal-to 0.06 as a small effect, 0.06 to less-than 0.14 as a medium effect, and greater-than or equal-to 0.14 as a large effect [60].

## 3. Results

At baseline, 21 out of 60 women (ECAU: *n* = 10; RISE: *n* = 11; 35% of total sample) screened positive for IPV-related TBI history. There were no significant differences between women with an IPV-related TBI history and women without this TBI history on age, race, ethnicity, sexual orientation, marital status, military branch, and length of time in relationship with abusive partner (see Table 1 for descriptive statistics). However, regarding whether they were still in a relationship with the abusive partner that prompted help-seeking, women with an IPV-related TBI history were significantly more likely to have left the relationship, χ^2^(N = 60) = 4.40, *p* = 0.036. Only 3 out of 21 women (14.3%) with a history of IPV-TBI were still in a relationship with this intimate partner, in comparison to 14 of 39 (35.9%) of women without this TBI history. Further, at baseline, women with IPV-related TBI status had significantly higher PTSD symptom severity (*M* = 47.1, *SD* = 16.03) than women without IPV-TBI (*M* = 37.17, *SD* = 16.21), *t*(51) = −2.71, *p* = 0.017. There was not a significant difference in depressive symptoms at baseline between the two groups.

At baseline, women with a history of IPV-related TBI experienced significantly more types of physical IPV (*M* = 5.45, *SD* = 4.07), *t*(25.63) = −3.66, *p* = 0.001, and sexual IPV (*M* = 2.25, *SD* = 2.57), *t*(27.71) = −2.25, *p* = 0.033, than women without this TBI history (*M* = 1.85, *SD* = 2.17; *M* = 0.82, *SD* = 1.57, respectively). As there was a significant difference between the TBI status groups in terms of ongoing involvement with their abusive partner, we included this variable as a covariate in our analyses. Subsequently, a repeated-measures ANCOVA revealed a significant interaction effect of IPV-related TBI history and time on the number of types of physical IPV experiences, *F*(1.76, 86.33) = 10.79, *p* < 0.001, η = 0.18 (large effect), such that women with histories of IPV-related TBI experienced a significantly accelerated decrease in these experiences from baseline to the follow-up sessions (see Table 2 for ANCOVA results) across both RISE and ECAU. A second repeated-measures ANCOVA showed a significant interaction effect of IPV-related TBI history, time, and treatment condition on the number of types of sexual IPV experiences, *F*(1.45, 70.83) = 3.93, *p* = 0.036, η = 0.07 (medium effect). Women with a history of IPV-related TBI history had significantly more types of sexual IPV experiences than women without this TBI history at baseline in the ECAU condition, and this same disparity was found for the 14-week follow-up for women in the RISE condition. However, the main effect of time was significant as well, indicating that women with IPV-related TBI histories in both treatment conditions experienced a significant decline in sexual IPV victimization, despite the disparity at 14 weeks for the RISE participants.

## 4. Discussion

This study aimed to explore the impact of IPV-related TBI history on safety outcomes (i.e., physical and sexual IPV revictimization) following a brief, modular-based IPV intervention, RISE, and its comparison condition, advocacy-based ECAU. Results from this secondary analysis of an RCT suggest that both RISE and ECAU are effective in improving safety outcomes for women with IPV-related TBI histories. Our hypotheses were partially supported, in that both treatments significantly reduced physical IPV revictimization experiences among women with IPV-related history in comparison to women without a history of IPV-related TBI. Interestingly, however, there was a significant difference in the number of types of sexual IPV revictimization experiences at baseline for the ECAU condition and at the 14-week follow-up for the RISE condition, with IPV-related TBI survivors experiencing more sexual IPV at both time points in their respective treatment conditions. Although this finding was unexpected, all women in the study experienced significant reductions in sexual IPV over the course of treatment and follow-up. These physical and sexual IPV results indicate that these brief interventions are safe for women with IPV-related TBI histories and may help sustain their long-term well-being in terms of attenuating the risk of revictimization.

Another notable finding was that over one-third (35%) of women in this sample screened positive for IPV-related TBI. These results are consistent with previous research, which generally reports IPV-related TBI rates ranging between 33% and 75% among women experiencing IPV [25,26]. Further, this finding aligns with the prevalence of IPV-related TBI in a community-based sample of IPV survivors with TBI assessed with a clinician-administered IPV-related TBI assessment [16,25], as well as prior research with women Veterans who have experienced IPV [55]. Despite the concordance between the prevalence of IPV-related TBI found in the present study and previous research, it is important to acknowledge the wide variability in positive screening for IPV-related TBI shown among the literature. This wide range in IPV-related TBI prevalence is likely due to inconsistent operational definitions and/or a lack of consistent and comprehensive screening and measurement tools designed to assess IPV-related TBI exposure across these various studies. Notably, the sample of treatment-seeking women who experienced past-year IPV examined in the present study reported a lower prevalence of IPV-related TBI compared to other samples of IPV survivors, which have been reported at up to 100% of the sample [24,26]. This discrepancy between IPV-related TBI prevalence across studies highlights the need for more consistent screening and validated screening tools for IPV-related TBI, which can determine prevalence and assess health-related outcomes.

Regarding safety outcomes, at baseline, women with histories of IPV-related TBI in this sample were more likely to have left their abusive relationship and to have significantly higher severity of PTSD symptoms in comparison to women without IPV-related TBI. This was likely due to the higher amount of physical and sexual IPV experiences for this group at this timepoint. Although reasons for leaving abusive relationships are complex, fear of physical injury and/or death has been cited as a significant consideration for women [29,61,62]. Thus, the fear associated with experiencing serious physical violence among women who experienced IPV-related TBI may have contributed to this finding. Women with histories of IPV-related TBI experienced significant reductions in physical and sexual IPV experiences across both interventions. This finding is new, and as such addresses the gap in knowledge about the impact of IPV-related TBI on treatment and recovery outcomes among women who have experienced IPV. Specifically, results suggest that brief healthcare-based interventions may enhance safety among women with histories of IPV-related TBI. Given that findings from the parent RCT demonstrated that both interventions were associated with reductions in general distress [46], the current finding expands upon research showing reductions in PTSD and depression are associated with lower risk for revictimization [63]. This is important, because a prior study found that a history of IPV-related TBI was associated with increased risk of mental health symptoms and future IPV [27]. It is possible that brief IPV interventions implemented within the healthcare setting may help break this cycle of IPV and distress among women with and without a history of IPV-related TBI. Although the responsibility for IPV revictimization always belongs to those who use the violence, identifying markers of IPV-related distress that can be directly targeted for intervention with potential downstream effects to reduce revictimization is of paramount importance for women experiencing IPV-related TBI. These efforts could substantially enhance safety and well-being for these women, given their high risk of exposure to severe violence and potential devastating effects (e.g., cognitive impairment, disability, death). Further work examining the impact of IPV-related TBI on broader psychosocial outcomes (e.g., mental health symptoms, empowerment, self-efficacy) from these types of interventions would provide additional context for these safety-related outcomes, and further benefit the field.

This study should be considered with its limitations, which include modest sample size, lack of a time- and attention-matched control condition, and the relatively brief interval for follow-up assessments, which precluded examination of longer-term safety outcomes. There is also the potential for recall bias from self-reported data, which may result in under- or over-reporting of probable IPV-related TBI history. Additionally, IPV-related TBI status in this analysis was assessed with a positive self-report screen, rather than a full assessment (such as the BATL-IPV) [25], limiting its ability to fully assess TBI status. It is unknown how recent IPV-related TBIs occurred, the number of TBIs experienced (from IPV and other experiences such as sports), or whether they occurred at the hands of the partner that led them to seek treatment [64]. Moreover, aspects of TBI such as post-traumatic amnesia or dissociation in response to a traumatic situation often make self-reported injury data challenging to accurately recall. These are important contextual factors that should be examined in future research. Future work using more comprehensive lifetime TBI assessments would ensure accuracy of TBI status and enable more nuanced understanding of the possible impact of IPV-related TBI on treatment outcomes among women who experience IPV. Additionally, this study is a secondary analysis of data from a larger RCT. The RCT was designed to examine the relative effectiveness of RISE on patient psychosocial health outcomes, but it was not designed or powered specifically for examining safety outcomes with respect to IPV-related TBI status. Additional trials with larger samples that are fully powered to detect differences by IPV-related TBI status would increase the reliability of our results. Further, research with follow-up assessments completed over longer time periods would better evaluate long-term impacts of treatment on safety outcomes. Long-term safety is especially relevant for this group, given the often-ongoing nature of IPV, which can perpetuate chronically. Given the health and safety benefits of the healthcare-based interventions in this analysis, future research should examine effectiveness with broader populations. These include people with diverse gender identities (e.g., non-binary, transgender) beyond cisgender women. This work could also evaluate potential benefits and feasibility in contexts other than within VHA settings and help ensure all people experiencing IPV-related TBI have access to effective interventions.

## 5. Conclusions

In sum, women who experience IPV-related TBI are often involved in a cycle of abuse which can persist on a chronic basis. Although the prevalence and impacts of IPV-related TBI have been increasingly recognized in research, much work still needs to be carried out to effectively identify IPV-related TBI and address the associated wide-reaching health and safety concerns in both research and practice. Our findings demonstrating significant reductions in physical and sexual violence among women with IPV-related TBI reinforce the value of healthcare interventions to improve safety for this population. These safety-related outcomes are especially important for women with histories of IPV-related TBI, given they often repeatedly experience severe physical violence. This study highlights the continued need for implementation of promising IPV-focused interventions to promote safety and protect women from experiencing further IPV.

## Figures and Tables

**Table 1:** Descriptive Statistics by IPV-related TBI Status.

Sociodemographic Characteristic	IPV-Related TBI (*n* = 21)	No IPV-TBI Hx (*n* = 39)	Statistic	*p* Value
Age, mean (SD)	36.4 (8.9)	40.5 (13.2)	*t* = 1.42	0.16
Race			χ^2^ = 2.62	0.45
Black	4 (19.0)	9 (23.1)		
White	12 (57.1)	21 (53.8)		
Asian	1 (4.8)	1 (2.6)		
Mixed Race	2 (9.5)	5 (12.8)		
Other Race	2 (9.5)	3 (7.7)		
Ethnicity (Hispanic)	4 (19.0)	8 (20.5)	χ^2^ = 0.02	0.89
Sexual Orientation			χ^2^ = 1.04	0.79
Heterosexual	14 (66.7)	29 (74.4)		
Lesbian/Gay	1 (4.8)	3 (7.7)		
Bisexual	4 (19.0)	5 (12.8)		
Pansexual	2 (9.5)	2 (5.1)		
Marital Status			χ^2^ = 4.55	0.47
Married/Living Together	3 (14.3)	9 (23.1)		
Married/Not Living Together	6 (28.6)	5 (12.8)		
Not Married/Not Living Together	1 (4.8)	3 (7.7)		
Living Together/Not Married	3 (14.3)	9 (23.1)		
Single	5 (23.8)	11 (28.2)		
Separated	3 (14.3)	2 (5.1)		
Ongoing Involvement with Violent Partner			χ^2^ = 4.40	0.036
Yes	3 (14.3)	14 (35.9)		
No	18 (85.7)	20 (51.3)		
Military Branch			χ^2^ = 7.71	0.17
Army	15 (71.4)	16 (41.0)		
Navy	0 (0)	7 (17.9)		
Air Force	3 (14.3)	5 (12.8)		
Marine Corps	3 (14.3)	6 (15.4)		
Reservists	0 (0)	1 (2.6)		
National Guard	0 (0)	2 (5.1)		
Physical IPV, mean (SD)				
Baseline	5.45 (4.07)	1.85 (2.17)		
10-Week F/U	0.80 (2.31)	0.76 (1.48)		
14-Week F/U	0.65 (2.23)	0.39 (0.90)		
Sexual IPV, mean (SD)				
Baseline	2.25 (2.57)	0.82 (1.57)		
10-Week F/U	0.70 (1.49)	0.18 (0.58)		
14-Week F/U	0.60 (1.88)	0.15 (0.44)		

Note. TBI = Traumatic Brain Injury; Hx = History; IPV = Intimate Partner Violence; F/U = Follow-Up.

**Table 2:** Repeated-Measures ANCOVA Results.

Physical IPV											
Time			Time × TBI			Time × Condition			Time × TBI × Condition		
*F*	*p*	η	*F*	*p*	η	*F*	*p*	η	*F*	*p*	η
33.06	<0.001	0.40	10.79	<0.001	0.18	1.22	0.297	0.02	1.02	0.356	0.02
**Sexual IPV**											
**Time**			**Time × TBI**			**Time × Condition**			**Time × TBI × Condition**		
***F***	***p***	**η**	***F***	***p***	**η**	***F***	***p***	**η**	***F***	***p***	**η**
14.29	<0.001	0.23	2.60	0.097	0.05	1.25	0.283	0.025	3.93	0.036	0.074

Note. IPV = Intimate Partner Violence; TBI = Traumatic Brain Injury; Condition = Treatment Condition (i.e., RISE or ECAU).

## Data Availability

The data that support the findings of this study are available upon reasonable request via a Data Use Agreement. The data are not publicly available due to privacy restrictions.

## List of Abbreviations

**Abbreviation**	**Meaning**
ANOVA	Analysis of Variance
ANCOVA	Analysis of Co-Variance
CES-D	Center for Epidemiological Studies Depression Scale
CTS2	Conflict Tactics Scale-Revised
ECAU	Enhanced Care as Usual
IPV	Intimate Partner Violence
PTSD	Post-Traumatic Stress Disorder
RCT	Randomized Clinical Trial
RISE	Recovering from IPV through Strengths and Empowerment
TBI	Traumatic Brain Injury
VA	Veterans’ Affairs
VHA	Veterans Health Administration
